# Carnitine Deficiency in OCTN2^−/−^ Newborn Mice Leads to a Severe Gut and Immune Phenotype with Widespread Atrophy, Apoptosis and a Pro-Inflammatory Response

**DOI:** 10.1371/journal.pone.0047729

**Published:** 2012-10-24

**Authors:** Srinivas Sonne, Prem S. Shekhawat, Dietrich Matern, Vadivel Ganapathy, Leszek Ignatowicz

**Affiliations:** 1 Department of Pediatrics, Medical College of Georgia, Georgia Health Science University, Augusta, Georgia, United States of America; 2 Department of Biochemistry and Molecular Biology, Medical College of Georgia, Georgia Health Sciences University, Augusta, Georgia, United States of America; 3 Departments of Laboratory Medicine & Pathology, Medical Genetics, and Pediatric & Adolescent Medicine, Mayo Clinic College of Medicine, Rochester, Minnesota, United States of America; 4 Center for Biotechnology and Genomic Medicine, Medical College of Georgia, Georgia Health Sciences University, Augusta, Georgia, United States of America; Charité, Campus Benjamin Franklin, Germany

## Abstract

We have investigated the gross, microscopic and molecular effects of carnitine deficiency in the neonatal gut using a mouse model with a loss-of-function mutation in the OCTN2 (SLC22A5) carnitine transporter. The tissue carnitine content of neonatal homozygous (OCTN2^−/−^) mouse small intestine was markedly reduced; the intestine displayed signs of stunted villous growth, early signs of inflammation, lymphocytic and macrophage infiltration and villous structure breakdown. Mitochondrial β-oxidation was active throughout the GI tract in wild type newborn mice as seen by expression of 6 key enzymes involved in β-oxidation of fatty acids and genes for these 6 enzymes were up-regulated in OCTN2^−/−^ mice. There was increased apoptosis in gut samples from OCTN2^−/−^ mice. OCTN2^−/−^ mice developed a severe immune phenotype, where the thymus, spleen and lymph nodes became atrophied secondary to increased apoptosis. Carnitine deficiency led to increased expression of CD45-B220^+^ lymphocytes with increased production of basal and anti-CD3-stimulated pro-inflammatory cytokines in immune cells. Real-time PCR array analysis in OCTN2^−/−^ mouse gut epithelium demonstrated down-regulation of TGF-β/BMP pathway genes. We conclude that carnitine plays a major role in neonatal OCTN2^−/−^ mouse gut development and differentiation, and that severe carnitine deficiency leads to increased apoptosis of enterocytes, villous atrophy, inflammation and gut injury.

## Introduction

Neonatal necrotizing enterocolitis (NEC) is a potentially fatal gastrointestinal emergency of the neonate. It is an acute intestinal necrosis syndrome of unknown etiology and occurs primarily among prematurely delivered neonates [1]. Its overall incidence has been reported to be around 0.3 to 2.4 cases per 1000 live births and it accounts for 2–5% of all NICU admissions and affects 5–25% of extremely low birth weight infants (<29 weeks gestation) [2]. Improved standards of care in the NICU have resulted in more preterm survivors; consequently more cases of this potentially fatal illness are reported [3]. The overall mortality related to NEC continues to be 20–30% and is inversely proportional to the gestational age, approaching 40–50% in extremely low birth weight neonates. NEC, especially in surgical cases leads to significantly prolonged hospitalization and long-term neurodevelopmental impairment [4], [5], [6]. Overall up to 45% of NEC survivors suffer from neurodevelopmental delay and cause significant increase in health care costs [7].

Despite several decades of research, the pathogenesis of NEC is still poorly understood. An interplay of several risk factors, which include intestinal ischemia-reperfusion injury, enteral feeding, and poor host defenses leading to sepsis, have been implicated; but prematurity and feeding are the two accepted, most important risk factors for this illness [3], [8]. Infant formulas have been modified to reduce osmolality and several additives have been considered to help reduce NEC occurrence, however, over the years only breast milk has been proven to be clearly beneficial [9]. Thus nutrition plays an important role in its causation but the exact role played by individual macro- and micronutrients is still not delineated.

Carnitine (β-hydroxy γ-trimethylaminobutyrate) is a conditionally essential nutrient. It is obligatory for transport of long-chain fatty acids into mitochondria for their subsequent β-oxidation. Therefore, carnitine plays a critical role in energy metabolism of tissues that derive a substantial portion of their metabolic energy from fatty acid oxidation. Such tissues in the past included the heart, skeletal muscle, liver, and placenta, and recently several reports have elucidated its role in the GI tract [10], [11], [12]. The biological importance of carnitine is underscored by the severe clinical consequences of carnitine deficiency seen in humans [13], [14].

Two distinct types of carnitine deficiency have been identified. Primary carnitine deficiency arises from defects in the plasma membrane carnitine transporter OCTN2. Patients with this disorder excrete carnitine in urine due to defective reabsorption, and plasma and tissue levels of carnitine drop below 10% of normal [15], [16], [17], [18], [19]. These patients have marked defects in fatty acid oxidation. Secondary carnitine deficiency arises from defects in any of the enzymes involved in fatty acid oxidation. In patients with these disorders, organic acids accumulate due to defective fatty acid oxidation and these organic acids enhance urinary excretion of carnitine in the form of acylcarnitines [10], [20]. Both forms of carnitine deficiency are associated with severe clinical consequences, notably hypoglycemia, cardiomyopathy, skeletal myopathy, arrhythmias, neuropathy, fatty liver, and in some cases sudden unexpected death [21], [22].

**Table 1:** Primers used for RT-PCR studies of FAO enzymes.

Accession Number	Enzyme	Amplicon (bp)	Sense primer	Antisense primer
NM_178878	LCHAD	520	CAA CGA CCA AAT CAG GAG TG	AGA GAC TTT CCG ATC AGC C
NM_007381	LCAD	576	CCA CTC AGA TAT TGT CAT GCC C	ACC ATT TCC CCC CCT TTT CC
NM_145558	LKAT	564	GGA AAG GAC ACA GTT ACC AAA G	TGA CAC AGA CAG GAA TAA GGA G
NM_017366	VLCAD	584	CCA CCA GAG AAA AAC CAG CC	AGA ATA GCC ATC CGA GCC AG
NM_007382	MCAD	610	AAG ACC AAA GCA GAG AAG AAG	CAT TGT CCA AAA GCC AAA CC
NM_008212	SCHAD	540	GGA CCA AAC GGA AGA CAT C	GGA CTG GGC TGA AAT AAG GG
NM_013556	HPRT	176	GCG TCG TGA TTA GCG ATG ATG AAC	CCT CCC ATC TCC TTC ATG ACA TCT

Most of the studies reported so far on carnitine deficiency in the neonate focus on the clinical consequences due to dysfunction of heart and skeletal muscle; little attention has been paid to its effects on the gastrointestinal tract. Whenever a fatty acid oxidation disorder (FAOD) is diagnosed, most are promptly treated with carnitine supplementation, and GI pathology escapes attention of most clinicians. Carnitine’s role in the GI tract has recently been highlighted by several publications linking mutations in genes encoding carnitine transporters OCTN1 (SLC22A4) and OCTN2 (SLC22A5) with Crohn’s disease (CD) [23], [24], [25], [26], [27], [28], [29]. Patients with CD have been shown to have a missense substitution 1762C→T in OCTN1 which causes amino acid substitution L503F and a G→C transversion in the promoter region of OCTN2 (−207G→C), which disrupts a heat shock binding element (HSE) in the promoter region of OCTN2 gene. These mutations lead to decrease in plasma membrane transport of carnitine and thus reduce tissue content of carnitine. Carnitine has also been shown to play a protective role in hypoxia/reoxygenation injury in the neonatal mice [30], [31] but there is paucity of information regarding its effects on developing gut and immune function in the neonatal GI tract.

**Table 2:** Tissue carnitine content of small intestine, spleen and thymus of OCTN2 mice and total lymphocyte count of spleen, thymus and lymph nodes isolated from each mouse (n = 6).

Tissue Carnitine content (nmoles/mg)				Total Lymphocyte count (×10^6^/mm^3^)		
	Small Intestine	Spleen	Thymus	Spleen	Lymph node	Thymus
OCTN2^+/+^	9.2±1.9	11.04±2.9	9.82±1.98	40.2±2.2	19.6±1.9	19.8±1.0
OCTN2^−/−^	2.1±0.2	0.12±0.01	0.2±0.1	4.06±0.8	2.47±0.9	3.53±0.8
P value	.004	<0.003	<0.003	0.003	0.01	0.03

Preterm infants are born with 40–50% lower plasma and tissue levels of carnitine [32], [33], [34], [35]. The normative levels and postnatal changes in plasma carnitine in the preterm neonate are now well characterized [36], [37], [38]. Sick preterm neonates who do not receive carnitine supplementation via the enteral or parenteral route continue to have lower tissue carnitine levels [39], [40], [41], [42] which drop further, and thus may develop a state of relative tissue carnitine deficiency. We hypothesize that tissue carnitine deficiency will have an impact at the molecular level in the developing GI tract. In this report, we have investigated the role played by carnitine in the neonatal gut using a mouse model of carnitine deficiency due to defective OCTN2.

## Materials and Methods

### Animals and Sample Preparation

We obtained a breeding pair of heterozygous OCTN2^+/−^ mice from Prof. Ikumi Tamai, Kanazawa University, Japan. Heterozygous OCTN2^+/−^ mice are viable and fertile so several pairs of OCTN2^+/−^ heterozygous males and females were mated, pregnant OCTN2^+/−^ mice were allowed to deliver and the pups were observed closely after birth. Homozygous OCTN2^−/−^ and age-matched wild-type OCTN2^+/+^ mice in the litters were genotyped as described earlier [43]. Wild type (OCTN2^+/+^) and homozygous (OCTN2^−/−^) pups were sacrificed around 7 days of life to collect tissue samples and to identify the various GI tract and immune system pathologies evident by this time. The experimental procedures were approved by the Institutional Animal Care and Use Committee of the Medical College of Georgia, Georgia Health Sciences University, GA, USA.

### Semi-quantitative RT-PCR for Enzymes Involved in β-oxidation

Primers were designed using Oligo® primer analysis software 6.0 (National Biosciences Inc. Cascade, CO, USA) for 6 enzymes involved in β-oxidation of fatty acids. Primer sequences and amplicon size for each enzyme are as shown in Table-1, all assays were carried out as described earlier [12]. Densitometry was performed using a SpectraImager 5000 Imaging system and AlphaEase 32-bit software (Alpha Innotech, San Leandro, CA, USA). Each experiment was repeated at least 6 times in each group from different mouse samples.

**Figure 1:**
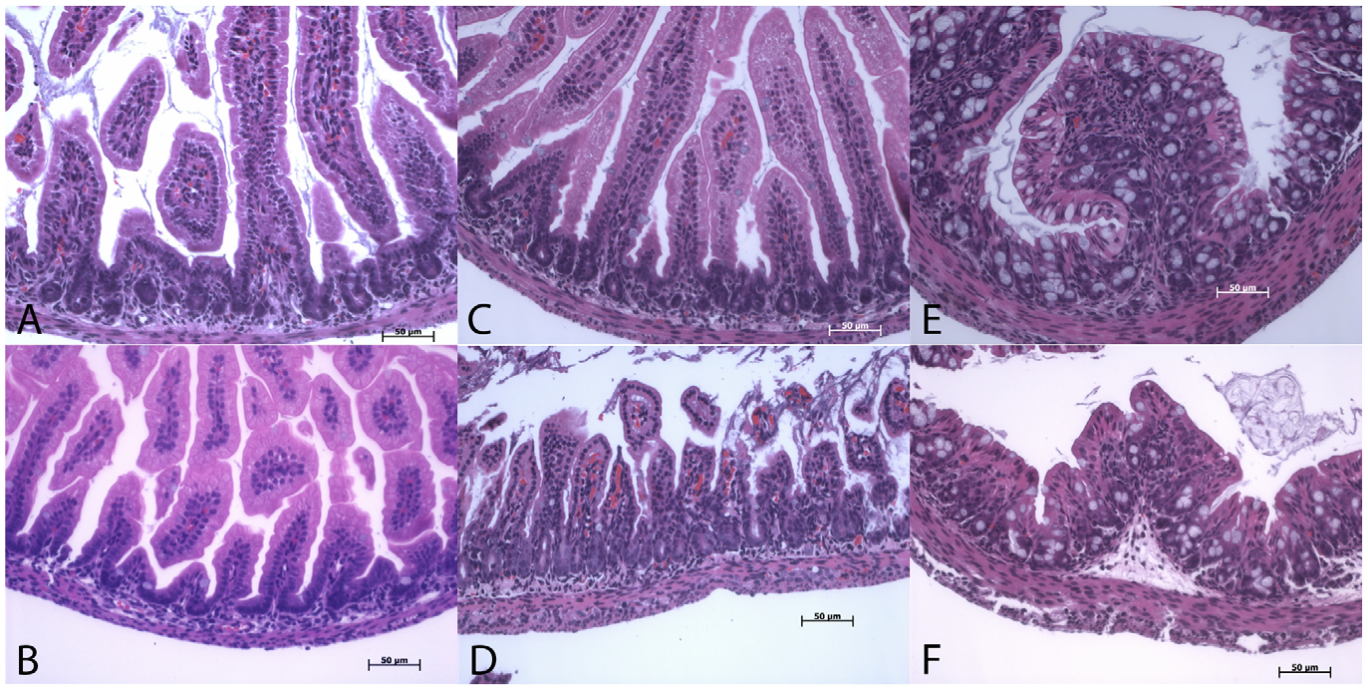
Gut histology of 1-week-old wild type (OCTN2^+/+^) and age-matched homozygous (OCTN2^−/−^) mice (H&E staining, X20). Images A–C represent photomicrographs of wild type (OCTN2^+/+^) jejunum, ileum and colon respectively and images D–F represent photomicrographs of homozygous (OCTN2^−/−^) jejunum, ileum and colon at the same magnification. (Bar represents 50 µm).

**Figure 2:**
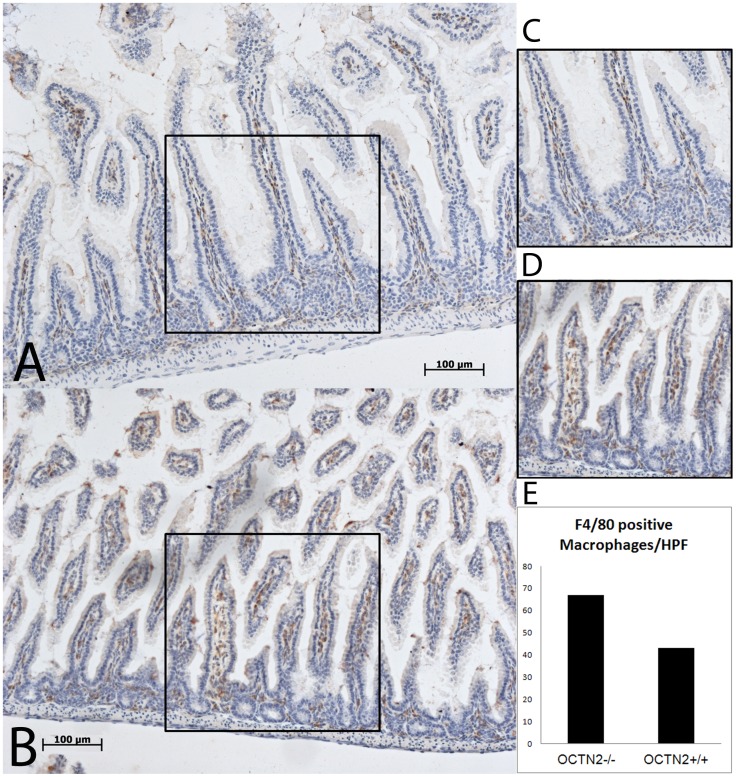
Immunohistochemical analysis of macrophage infiltration in 1-week-old wild type (OCTN2^+/+^) and homozygous (OCTN2^−/−^) mice ileum sections. Panel A is an ileum section showing F4/80 staining (brown) of villous macrophages from the wild-type mouse and panel C is a high power magnification of a representative area to help count the number of F4/80 positive cells. Panel B is an ileum section of a homozygous mouse and panel D is a high power magnification of a representative area. Panel E is a graphic representation of the number of F4/80 positive cells in each section (Bar represents 100 µm).

### Histopathology and Immunohistochemistry of Gut Samples

Mouse gut samples were fixed in 10% formalin and 5–10 µm thick sections of paraffin-embedded tissue were cut, applied to glass slides, deparaffinized in xylene, and rehydrated in an ethanol gradient. A set of sections from 6 animals was stained with hematoxylin and eosin (H&E) for histological analysis. For immunohistochemistry, endogenous peroxidase activity was quenched by incubating the specimens in 3% H_2_O_2_ in methanol for 30 min and subjected to antigen retrieval.

The slides were then washed and blocked using an Avidin/Biotin blocking kit (Vector Labs, Burlingame, CA) for 30 min followed by a blocking buffer (NEN-Life Sciences, Boston, MA) for 30 min. The blocking buffer was removed, and the sections were exposed to primary rabbit polyclonal antibody specific for each of the following enzymes at the indicated dilution: MCAD (1∶200), LCAD (1∶400), VLCAD (1∶200), SCHAD (1∶200), LKAT (1∶400). (kind gift of Dr Arnold Strauss, Cincinnati Children’s Hospital, OH) and F4/80 (1∶1000) (Abcam Labs. Cambridge, MA). The staining was done as described earlier [12]. Sections were then incubated with Alexa Fluor 555–conjugated IgG (Invitrogen, Carlsbad, CA) secondary antibody (1∶1000). Slides were washed in PBS, incubated with a 1∶24,000 dilution of Hoechst stain, coverslipped, and viewed with an epifluorescence microscope (Axioplan-2 equipped with the Axiovision Program and an HRM camera; Carl Zeiss Meditec, Oberkochen, Germany). All immunostaining experiments were performed on at least 6 sets of gut tissue from 1-week-old mice for all enzymes and F4/80 positive cells.

**Figure 3:**
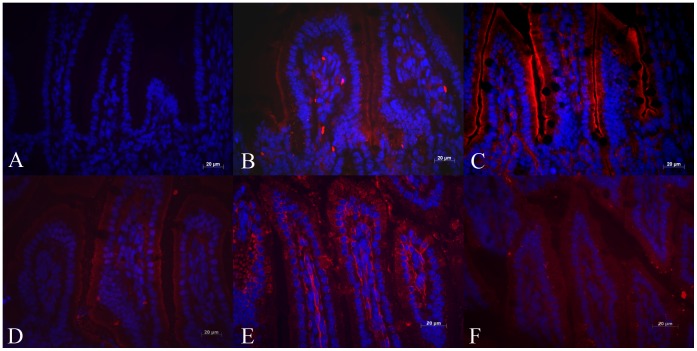
Immunohistochemical analysis of expression of fatty acid oxidation enzymes in 1-week-old wild type (OCTN2^+/+^) mouse jejunum. Panel A is a negative control image where there was no primary antibody and panels B–F shows immunoreactivity for MCAD, LCAD, VLCAD, SCHAD, and LKAT, respectively. All five enzymes are expressed in villous epithelial cells and minimal expression in non-epithelial cells of the villous core. (Bar represents 20 µm).

**Figure 4:**
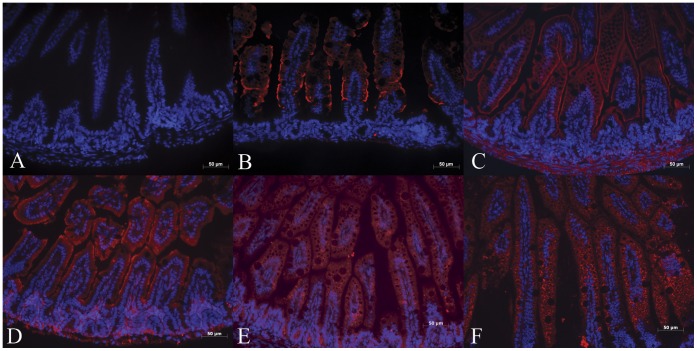
Immunohistochemical analysis of expression of fatty acid oxidation enzymes in 1-week-old wild type (OCTN2^+/+^) mouse ileum. Panel A is a negative control image where there was no primary antibody and panels B–F show immunoreactivity for MCAD, LCAD, VLCAD, SCHAD, and LKAT, respectively. All five enzymes are expressed mainly in the villous epithelial cells and minimal expression in non-epithelial cells of the villous core and crypts. (Bar represents 50 µm).

### Carnitine Analysis by Tandem Mass Spectrometry

Tissue samples from six OCTN2^+/+^ and age-matched OCTN2^−/−^ small intestine mucosal scrapings, spleen, and thymus were collected and protein concentrations of each sample was estimated by the Lowry method. The total, free and acyl-carnitine fractions were determined by tandem mass spectrometry as described [44] and results were expressed as carnitine content per mg of tissue.

**Figure 5:**
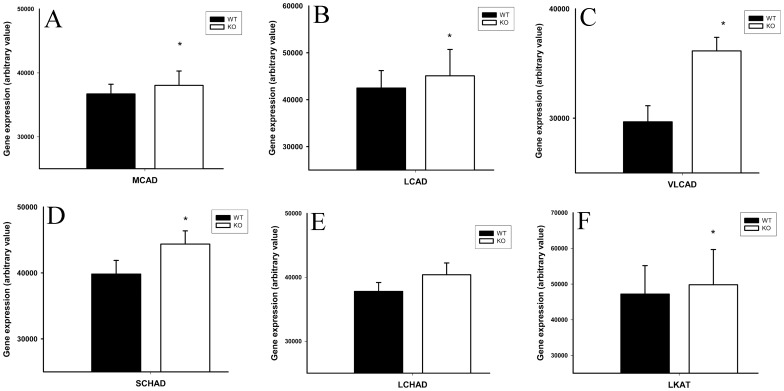
Composite image of semi-quantitative RT-PCR (densitometry) in small intestine mucosal scrapings for MCAD, LCAD, VLCAD, SCHAD, LCHAD and LKAT (Panel A to F) expression in 1-week-old OCTN2^+/+^ (black bar) and OCTN2^−/−^ mouse (white bar) respectively. Expression of HPRT was used to normalize data from each group. Asterisks (*) represents a statistically significant change in expression.

**Figure 6:**
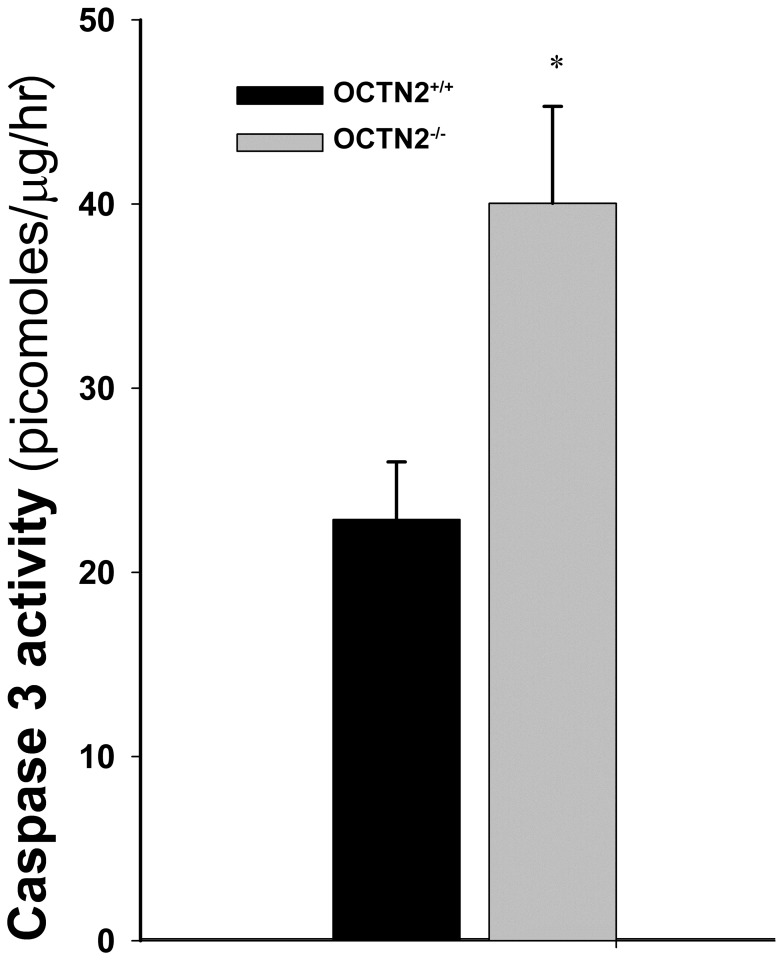
Caspase 3 activity in 1-week old small intestine mucosal scrapings from OCTN2^+/+^ (black bar) and OCTN2^−/−^ mice (white bar). Asterisk (*) represents statistically significant difference in activity.

### Apoptosis Assay in Gut, Spleen and Thymus Tissue

Activity of Caspase-3 was determined in small intestinal mucosal scrapings using a colorimetric method, CaspACE assay system™, (Promega, Madison, WI) per the manufacturer’s recommendations. To identify apoptosis in the spleen and thymus, the ApopTag *In Situ* Oligo Ligation (ISOL) technique was used with the T4 DNA ligase kit (Chemicon International, Temecula, CA) per the manufacturer’s recommendation.

### Flow Cytometry for Lymphocyte Subpopulation

Lymphocytes from spleen, thymus and lymph nodes were obtained by straining the gently crushed intact organ through a nylon mesh. Erythrocytes were lysed by treatment with an ammonium chloride solution. Cells were stained on ice. Viable cells were identified by gating on forward and side scatter, and CD4^+^, CD8^+^, CD24^+^ and CD45-B220^+^ cells were sorted using magnetic beads coated with individual antibody (MACS cell sorter, Miltenyi Biotec Inc. Auburn, CA, 95602, USA).

### Cytokine Bead Assay

The direct quantification of cytokines was done using a cytometric bead assay (CBA) (BD Biosciences, San Diego, CA) per the manufacturer’s recommendations. The assay is based on multiple flow cytometric bead-based immunoassays that measures different cytokines simultaneously, from a single cell culture supernatant. We used 3–4×10^5^ cells per well of total lymphocytes from OCTN2^−/−^ and OCTN2^+/+^mice. The dye, incorporated in the beads fluoresces strongly at 650 nm (measured as FL3 signals in BD FACScan and FACScalibur Flow Cytometers) when excited with an argon laser. Detection is mediated by the binding of specific detection antibodies that are directly conjugated with phycoerythrin (PE), to each of the corresponding capture bead/analyte complex populations, thus providing an FL2 fluorescent signal on the appropriate bead. This signal is proportional to the concentration of the cytokine in the test matrix. This kit combines beads with the ability to measure the levels of IL-2, IL-6, MCP-1, TNF-α and interferon-γ from the same sample. Cytokine concentration was measured by using established calibration curves and dedicated CBA analysis software.

**Figure 7:**
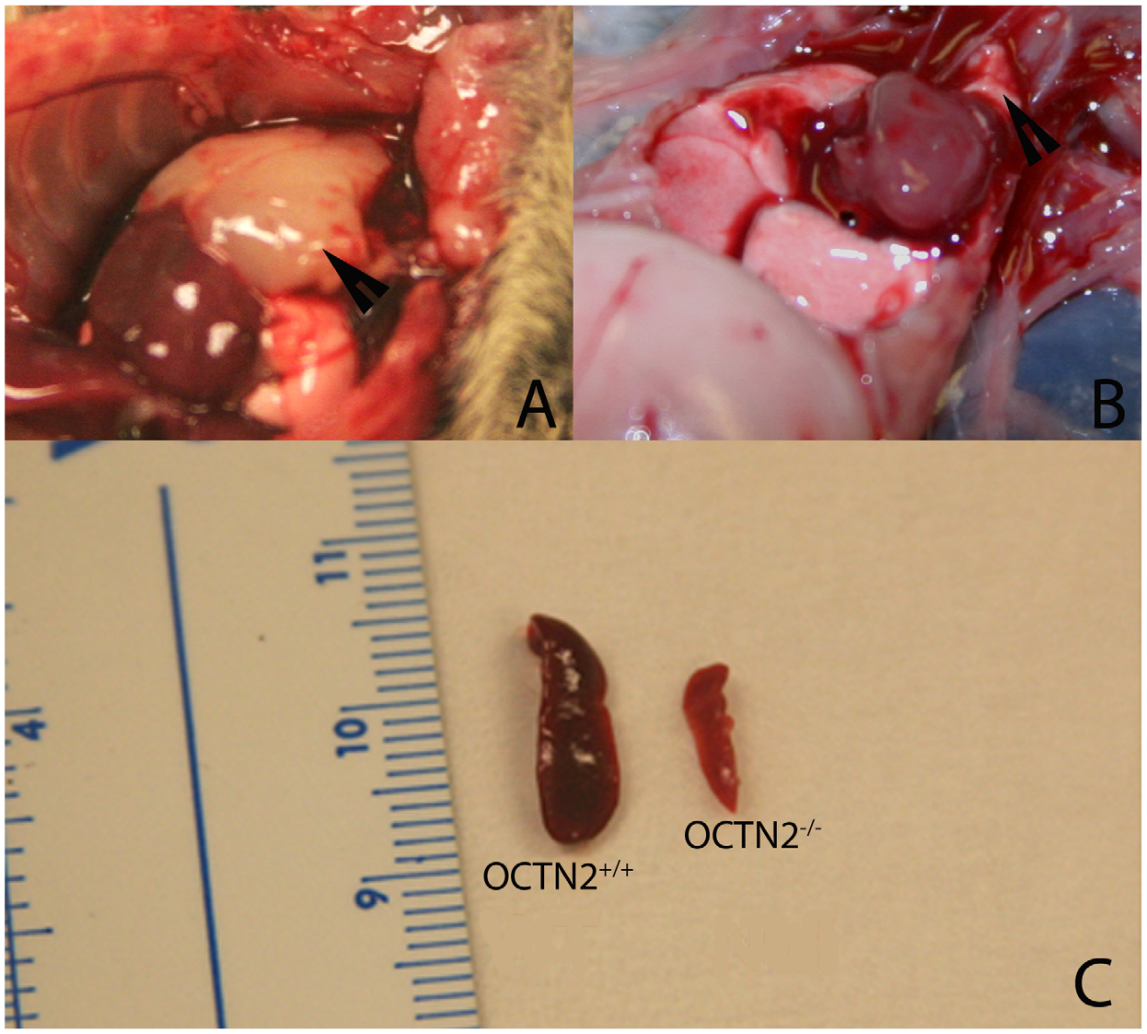
Gross appearance of thymus and spleen. Panel A is an image of OCTN2^+/+^ mouse thymus covering the heart (Arrowhead), and panel B is an image of OCTN2^−/−^ mouse thymus above the heart (Arrowhead). Panel C is an image of age-matched OCTN2^+/+^ (left) and OCTN2^−/−^ (right) mouse spleens.

**Figure 8:**
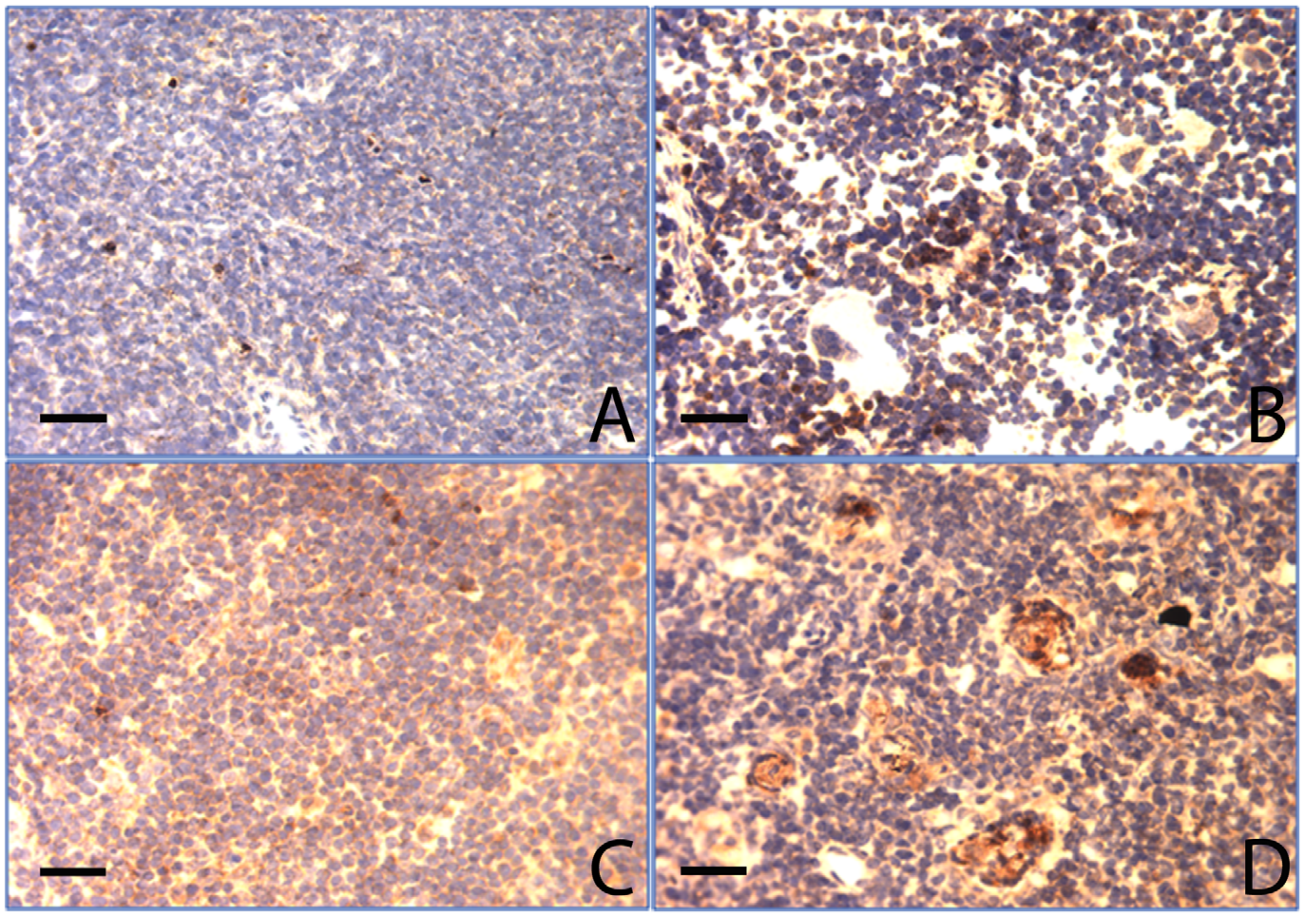
Analysis of apoptosis in thymus and spleen using Oligo-ApopTaq assay (×20). Panels A & B are OCTN2^+/+^ and OCTN2^−/−^ mouse spleen sections and panels C & D are OCTN2^+/+^ and OCTN2^−/−^ mouse thymus sections respectively. (Bar represents 50 µm).

### Realtime PCR for Expression of Genes Involved in Enterocyte Growth, Differentiation, and Migration

We used *RT^2^ profiler PCR arrays* (SABiosciences, Qiagen, Frederick, MD) to study differences in gene expression of *TGF-β/BMP pathways* (cat No. PAMM035) between small intestinal mucosal scrapings from 1-week-old OCTN2^−/−^ mice and age-matched wild type controls. RNA was extracted using the Trizol method from gut tissue from two sets of animals. This array is housed on a 96-well plate; it provides information on 84 genes which includes members of the TGF-β superfamily of cytokines and their receptors: SMAD and SMAD target genes, which include adhesion, extracellular molecules and transcription factors involved in downstream cellular processes. Details of this PCR array are available at the Qiagen website (http://www.sabiosciences.com/rt_pcr_product/HTML/PAMM-035A.html). Relative expression levels of each gene were analyzed using BioRad iQ iCycler Detection System (BioRad Laboratories, Hercules, CA, USA). All assays were performed per the manufacturers’ recommendations, each experiment was performed in duplicate, and results represented as the mean of each experiment.

### Statistical Analysis

All data are presented as means ± SD and comparisons between paired samples were made by Student’s‘t’ test with Bonferroni’s correction where applicable and statistical significance was set at p ≤ 0.05. We used statistical software SPSS for PC version 11.01 for data analysis.

## Results

The homozygous (OCTN2^−/−^) mice survive for about 4–5 weeks and look much smaller without carnitine supplementation compared to age-matched wild type (OCTN2^+/+^) littermates while they are indistinguishable when 1-week-old. The carnitine content in small intestine, spleen and thymus of neonatal OCTN2^−/−^ mouse is markedly lower than in the age-matched wild type OCTN2^+/+^ mouse (Table-2).

### 1-week-old OCTN2^−/−^ Mice Showed Signs of Poor Villous Growth and Differentiation throughout the GI Tract with Areas of Lymphocytic and Macrophage Infiltration

By the end of the first week of life the carnitine deficient OCTN2^−/−^ mice develop early signs of stunted villous growth in comparison to the wild-type mice (Fig. 1) and some areas of the gut show disruption of the villous architecture with lymphocytic infiltration and thickening of the muscular mucosa (Fig. 1, panel C & D). The colonic villi are not as healthy looking and the number of mucin producing goblet cells is markedly reduced (Fig. 1, panel E & F). F4/80 staining for macrophages showed a higher number of macrophages in the villous core of the OCTN2^−/−^ ileum sections (Fig. 2, panel B &D) compared to the age-matched wild-type controls (Fig. 2 panel A & C). The total number of macrophages in two respective high power field are quantified in Fig. 2, panel E.

### Five Key Enzymes Involved in Mitochondrial β-oxidation are Expressed in Enterocytes Lining the Small Intestinal Villi

There was high expression of MCAD, LCAD, VLCAD, SCHAD and LKAT in the enterocytes lining the villi of jejunum (Fig. 3) and ileum (Fig. 4) of 1-week-old wild-type (OCTN2^+/+^) mice. The enzyme expression (red fluorescence) was mainly localized to enterocytes over the villous tips with much less expression in villous core and villous crypts. There was complete absence of staining in negative control sections (Panel A, Fig. 3&4).

### Up-regulation of Genes of 6 Enzymes Involved in β-oxidation in 1-week-old OCTN2^−/−^ Mice

Fig. 5 is a composite image of semi-quantitative RT-PCR densitometry data for these enzymes. There was a significantly increased expression of MCAD, LCAD, VLCAD, SCHAD and LKAT genes in small intestinal mucosal scrapings from carnitine-deficient animals as compared to age-matched wild type (OCTN2^+/+^) mice.

### Carnitine Deficiency in OCTN2^−/−^ Mice Leads to Increased Apoptosis in the GI Tract

Activity of caspase 3 was significantly elevated in small intestinal mucosal scrapings from OCTN2^−/−^ animals compared to 1-week-old age matched wild-type controls (Fig. 6).

### OCTN2^−/−^ Mice Develop a Severe Immune Phenotype Characterized by Atrophy of Lymph Nodes, Thymus and Spleen

The wild-type (OCTN2^+/+^) mouse thymus filled up the whole of superior mediastinum and covered the heart (Fig. 7A, arrowhead) whereas the age-matched carnitine-deficient (OCTN2^−/−^) mouse thymus was small, shriveled and barely visible (Fig. 7B, arrowhead). Likewise the spleen of OCTN2^−/−^ mice was pale and much smaller in size (Fig. 7C). The total lymphocyte count from thymus, spleen and lymph nodes was much reduced (Table2). There was markedly increased lymphocyte apoptosis in the OCTN2^−/−^ mouse spleen and thymus as indicated by brown staining of lymphocytes with the Oligo Apoptaq assay. There were several “punched out” areas in the spleen and thymus sections where a complete loss of lymphocytes had occurred (Fig. 8). Flow cytometry studies showed that carnitine deficiency led to a change in relative percentage of lymphocyte surface markers. There was a relative decrease in CD4^+^ splenocytes in the OCTN2^−/−^ mouse and a marked increase in CD45-B220^+^ thymocytes and lymphocytes which indicates exposure to extrinsic environmental antigens and a T-cell response (Table-3).

**Table 3:** Relative populations of CD4^+^, CD8^+^, CD24^+^ and CD45-B220^+^ lymphocytes from spleen, thymus and lymph nodes of wild-type (OCTN2^+/+^) and homozygous (OCTN2^−/−^) mice (n = 6).

Genotype	Spleen				Thymus				Lymph node			
	CD 4	CD 8	CD 24	CD45-B220	CD 4	CD 8	CD 24	CD45-B220	CD 4	CD 8	CD 24	CD45-B220
OCTN2^+/+^	17.3±1.5	64.2±7.4	7.2±1.2	23.4±2.4	3.7±2.3	4.1±1.3	2.8±0.6	1.1±0.1	49.0±5.8	14.1±5.9	19.5±3.9	1.21±0.1
OCTN2^−/−^	26.8±2.7	55.8±4.3	10.1±1.5	25.5±2.1	7.0±4.7	6.4±2.3	3.8±2.5	40.9±2.2	50.9±7.8	14.5±4.2	14.5±1.6	18.2±1.8
P value	**0.01**	0.23	0.08	0.3	0.23	0.16	0.45	**<0.001**	0.65	0.82	0.11	**<0.001**

**Table 4:** Basal and anti-CD3 antibody stimulated cytokine production by lymphocytes from of wild-type (OCTN2^+/+^) and homozygous (OCTN2^−/−^) mice (n = 6).

Cytokine	Basal cytokine production (pg/ml)		Anti-CD3 stimulated cytokine production (pg/ml)	
	OCTN2^+/+^	OCTN2^−/−^	OCTN2^+/+^	OCTN2^−/−^
IL-2	4.98±1.1	18.4±1.5*	249±21.1	421±36.6*
IL-6	13.8±2.3	60.2±5.4*	600±54.2	880±65.4*
TNF-α	40.7±16.1	52.2±20.0	904±75.6	1706±122.8*
MCP-1	0.4±0.2	1.8±0.3	3909±245.6	4176±466.2
Interferon-γ	284±22.6	375±52.2	538±42.6	1030±82.4*

Asterisk (*) represents a statistically significant difference.

**Table 5:** Expression of genes involved in the TGF-β/BMP pathway in small intestine gut scrapings from wild-type (OCTN2^+/+^) mice relative to the expression pattern observed in homozygous (OCTN2^−/−^) mice (n = 2).

Accession No.	Molecule Name	Fold Change	Accession No.	Molecule Name	Fold Change
NM_009612	Activin A receptor II	−6.7	NM_019919	Latent TGF- binding protein 1	−4.0
NM_007554	Bone morphogenetic protein 4	−2.9	NM_175641	Latent TGF- binding protein 4	−5.3
NM_007556	Bone morphogenetic protein 6	−4.6	NM_008711	Noggin	−3.5
NM_007669	Cyclin-dependent kinase inhibitor 1A (P21)	−4.1	NM_007430	Nuclear receptor subfamily 0, group B, member 1	−3.4
NM_009930	Procollagen, type III, a1	−5.9	NM_008873	Plasminogen activator, urokinase	−4.9
NM_008343	Insulin-like growth factor BP-3	−3.5	NM_008539	MAD homolog 1 (Drosophila)	−3.9
NM_010580	Integrin beta 5	−7.2	NM_029438	SMAD specific E3 ubiquitin protein ligase 1	−3.6
NM_013566	Integrin beta 7	−2.7	NM_009283	Signal transducer and activator of transcription 1	−4.8
NM_008416	Jun-B oncogene	−5.9	NM_011577	Latent TGF- 1	−5.3
NM_009371	TGF- receptor II	−3.0	NM_009368	Latent TGF- 3	−3.1
NM_001013025	TGF- receptor associated protein 1	−4.0	NM_009369	Latent TGF- induced	−5.7

### OCTN2^−/−^ Lymphocytes Produce Increased Amounts of Pro-inflammatory Cytokines Under Basal and Stimulated Conditions

Splenocytes and thymocytes from OCTN2^−/−^ mice secreted significantly increased amounts of IL-2, IL-6, TNF-α, MCP-1 and IFN-γ under basal and anti-CD3 antibody-stimulated conditions (Table-4).

### Enterocytes of OCTN2^−/−^ Mouse Small Intestine have a Low Expression of TGF-β/BMP Pathway Genes

Table-5 summarizes the changes in expression of genes involved in the TGF-β/BMP signaling pathway. We found a similar trend in results of each of the two experiments and data shown represent mean values of the genes with >2 fold change. There was down-regulation of most TGF-β/BMP pathway genes and 22 genes showed a significant change. Genes for TGF-β 1 & 3, its receptor II, BMP 4 & 6, IGFBP3, integrin β 5 & 7, activin and noggin were significantly down-regulated in the OCTN2^−/−^ mouse gut.

## Discussion

Our previous investigations have demonstrated that carnitine deficiency leads to severe atrophy of the villous structure throughout the gut with widespread inflammation, perforation and abscess formation and signs of peritonitis in the adult OCTN2^−/−^ mouse [12]. In this report, we have explored the early effects of carnitine deficiency on the developing neonatal gut to investigate as to what role carnitine deficiency might play at the molecular level before gross changes are seen in the gut. The neonatal GI tract requires a constant supply of energy to support its own growth, maturation, nutrient transport function and maintenance of epithelial barrier function. This energy is produced by mitochondrial β-oxidation of long-chain fatty acids in enterocytes where carnitine plays a crucial role. We have earlier measured activity of two major fatty acid oxidation enzymes, long-chain L-3-hydroxyacyl CoA dehydrogenase (LCHAD) and short-chain L-3-hydroxyacyl CoA dehydrogenase (SCHAD) using adult OCTN2 mice and found that SCHAD and LCHAD activity in mouse gut is nearly 4 to 10 fold higher than in liver which is considered the major organ metabolizing fatty acids [12]. Our current data are in line with this finding and highlight the value of carnitine during the neonatal period. Carnitine’s role in the GI tract has recently been highlighted when several reports linked mutations in genes encoding carnitine transporters OCTN1 (SLC22A4) and OCTN2 (SLC22A5) with Crohn’s disease (CD) [23], [45]. Though carnitine’s immunosuppressive and therapeutic properties in gut inflammation have been described in the past [46], [47], [48], [49], [50], this is the first report about its role in development and differentiation of enterocytes and gut inflammation during the neonatal period. Carnitine’s role in gut associated immunity has recently been highlighted where it has been shown to abrogate gut inflammation and prevents lymphocyte apoptosis [46], [48], [51], [52]. Carnitine has also been used in adults with gut inflammation due to CD or ulcerative colitis with beneficial effects [53], [54], [55]. Carnitine deficiency in our mouse model causes a global effect on the immune system as evidenced by severe atrophy and apoptosis of splenocytes, thymocytes, lymph node lymphocytes as well as intra-epithelial lymphocytes. This was preceded by an early pro-inflammatory response in the gut with macrophage and lymphocytic infiltration. Thus OCTN2^−/−^ mouse spleen and thymus phenotypes are part of the same process.

Intestinal epithelial cells normally undergo apoptosis at a very high rate and apoptotic cells are quickly replaced by newer cells in a seamless manner. Any perturbation of this process leads to breach of the gut epithelial barrier and entry of gut pathogens into the blood stream. We have demonstrated a major increase in enterocyte apoptosis in our mouse model of carnitine deficiency which initiates this process of gut injury. Our earlier studies using the adult OCTN2^−/−^ have shown a higher expression of caspase 1 and 3 in small intestine by Western blot. We also found up-regulation of gut protective molecules such as phosphorylated ERK and AKT [12]. Several growth and transcription factors are required for the transformation of crypt stem cells into mature enterocytes and secretory cells and ensure integrity of gut mucosal barrier. These growth factors including the TGF-β/BMP are involved in various aspects of gut development and differentiation [56], [57], [58], [59], [60]. TGF-β/BMP act through their receptors and activate a number of intracellular SMAD transcriptional regulators [58], [60]. SMAD complexes interact with either co-activators or co-repressors of transcription to control target gene expression. Here we have presented data to show that TGF-β/BMP pathway genes are down-regulated in states of carnitine deficiency and thus will affect villous growth and differentiation. TGF-β/BMP pathway genes also regulate cytokine production and mucosal inflammation [61] and our studies in the OCTN2^−/−^ mouse show initiation and progression of immune response and increased pro-inflammatory cytokine production.

Results of our study, though conducted in an animal model, are relevant to cases of NEC, a disease which predominantly affects low birth weight preterm neonates since they are born with low tissue levels of carnitine and may not have received carnitine supplementation leading to an even lower tissue level of this conditionally essential nutrient. The heterozygous OCTN2^+/−^ mouse with lower tissue levels of carnitine develops a clinical phenotype comparable to the homozygous OCTN2^−/−^ mouse in adult life though its GI pathology has not been reported [62]. Nutrition plays a central role in causation of NEC as indicated by marked reduction in its incidence by use of human breast milk. More than 50% of calories in human breast milk are from fatty acids indicating that carnitine plays a significant role in the metabolism of these calories from fatty acids. The problem of low tissue carnitine levels in the preterm neonate may go unrecognized by physicians and may thus contribute to gut injury as commonly seen in NEC. Our animal model of carnitine deficiency is not a classic model of NEC where NEC is either created by using caustic agents like dextran sulphate or with LPS instillation in the stomach followed by subjecting animals to periodic hypoxia, but it does show the spontaneous changes in molecular events in the developing enterocytes which ultimately lead to gut injury. Thus our study supports the notion that carnitine supplementation is beneficial to preterm neonates who are at high risk of developing NEC.
